# Hand, foot, and mouth disease in adults caused by *Coxsackievirus B1-B6*^☆^

**DOI:** 10.1016/j.abd.2021.03.012

**Published:** 2022-03-07

**Authors:** Anama Di Prinzio, Dolores Pilar Bastard, Ana Clara Torre, Luis Daniel Mazzuoccolo

**Affiliations:** Department of Dermatology, Hospital Italiano de Buenos Aires, Buenos Aires, Argentina

**Keywords:** Adults, Enterovirus, Hand, foot and mouth disease

## Abstract

Hand, foot, and mouth disease is a viral rickettsial disease caused by *Coxsackievirus A16* and *Enterovirus* 71 in most cases. It is commonly seen in children under ten years old, who present oral enanthema and a macular, maculopapular, or vesicular rash on their hands and feet. However, an increase in cases caused by other viral serotypes was observed in adults in recent years with various clinical presentations and a troublesome diagnosis. Three cases of hand, foot, and mouth disease are reported to show the clinical variability and diagnostic complexity that this disease may present in adult patients.

## Introduction

Hand, Foot, and Mouth Disease (HFMD) is a rickettsial disease often caused by viruses of the *Picornaviridae* family, *Human Enterovirus* genus, within which *Coxsackievirus (CA) A16* and *Enterovirus (EV) 71* are the ones most frequently isolated. In 97% of cases, HFMD affects children under 10-years old. Serologic types A 5-7, A 10, B 1-3, and B5-B6 are sometimes isolated. According to literature, CA A6 is the most frequent serotype in adults.^1^, ^2^, ^3^, ^4^, ^5^, ^6^

HFMD has a high infection rate. Person-to-person transmission occurs through direct contact with nasal secretion, saliva, feces, or contaminated objects. The disease usually appears in the form of epidemic outbreaks in spring, summer, or early autumn. The incubation period is three to six days. After coming into contact, the virus is implanted in the oral or ileum mucosae spreading from there to the blood; this is known as primary viremia. After 24 hours in the blood, the virus starts spreading to lymphatic tissue and different organs. Respiratory elimination of the virus may persist for three weeks and digestive elimination for eight weeks.^2^, ^3^, ^4^, ^5^

In most cases, mainly in children, HFMD appears as enanthema, papules, and/or blisters on the hands and feet, which may resolve in one to two weeks.^2^

## Case Report

**Figure 1:**
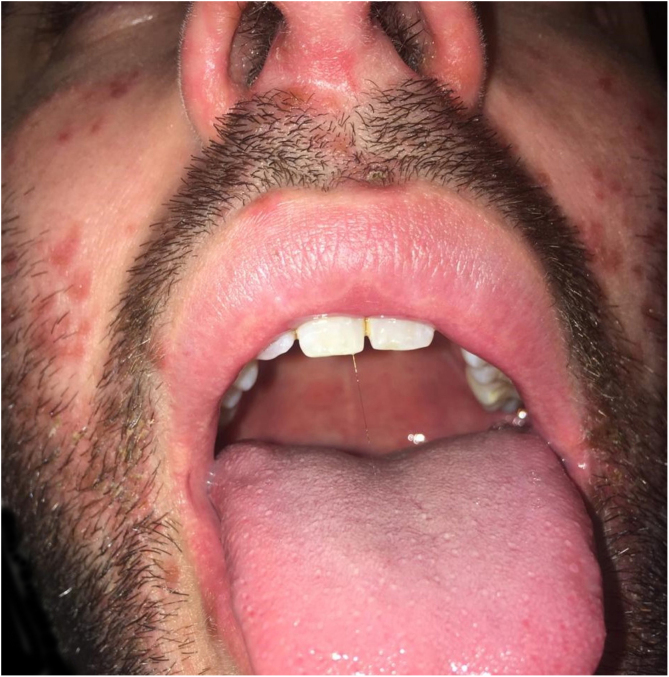
Oral enanthema.

**Figure 2:**
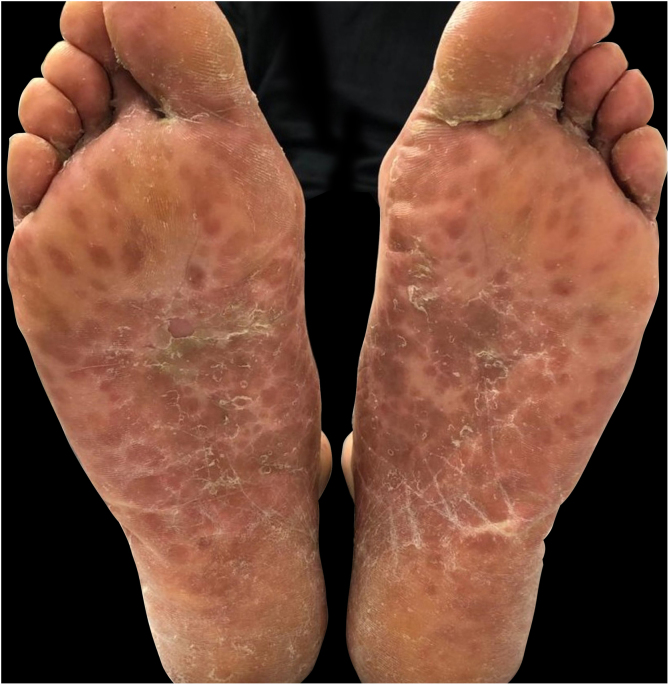
Erythematous purpuric macules and papules, and isolated blisters, located on the feet.

**Figure 3:**
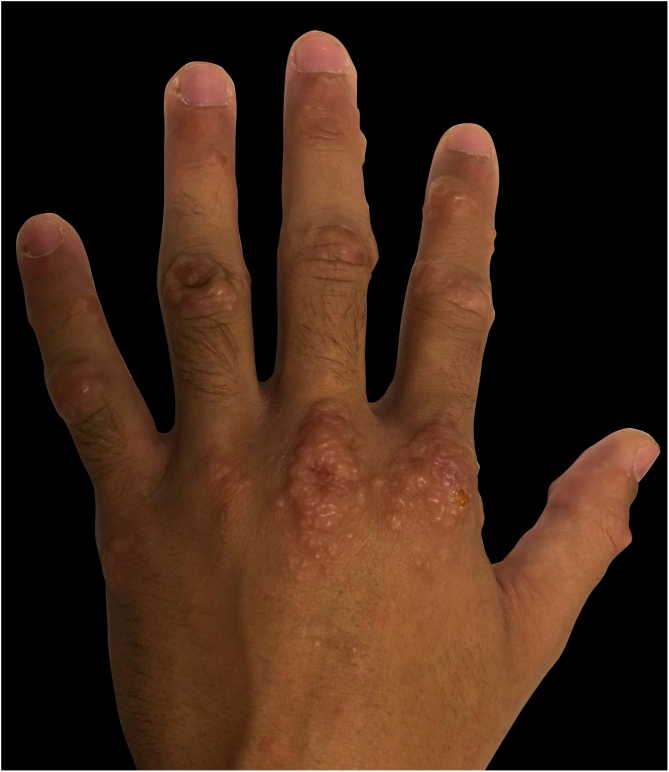
Multiple erythematous papules, with confluent areas, located on the hands.

**Figure 4:**
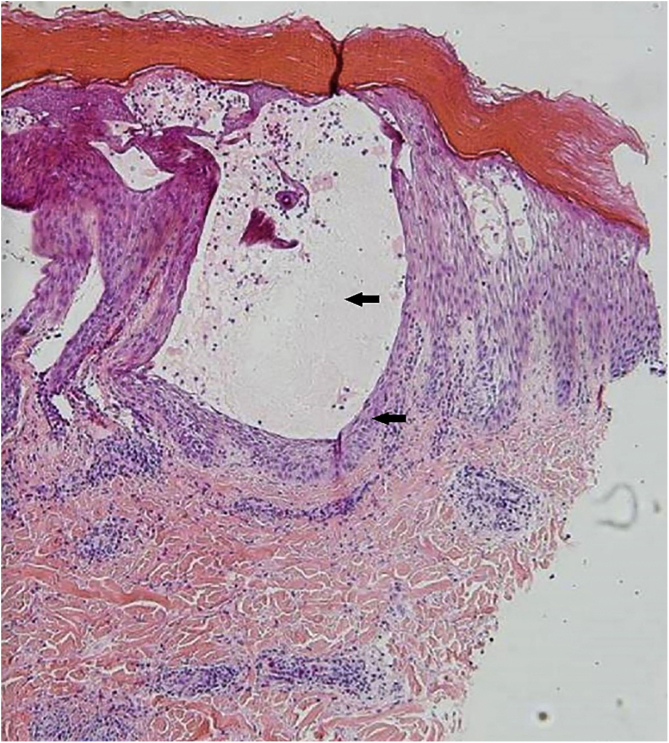
Skin biopsy on the arm showing intraepidermal blister and intense spongiosis (Hematoxylin & eosin, 40×).

**Figure 5:**
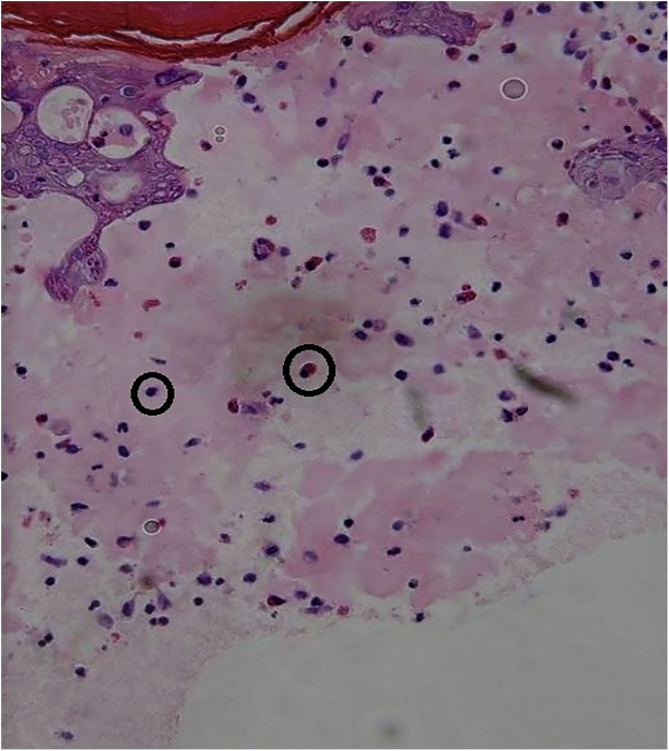
Serous blister formation with eosinophils and lymphocytes are observed. Edematous papillary dermis with mononuclear perivascular infiltration (Hematoxylin & eosin, 400×).

Three cases of HFMD are reported in adult patients. Its etiology, clinical manifestations, the differential diagnosis, the diagnostic approach, the treatment provided, and the clinical progress are described (Table 1) (Figure 1, Figure 2, Figure 3, Figure 4 and 5).

**Table 1:** Cases: Clinical record, clinical findings, laboratory, histopathology, diagnosis, treatment, and virus isolation.

	Patient 1	Patient 2	Patient 3
**Gender**	Male	Male	Male
**Age (years)**	33	39	21
**Epidemiological Background**	–	Daughter with HFMD, 1 week before	–
**Skin Lesions**	Oral enanthema and painful maculopapular rash	Multiple purpuric macules and papules, itchy and isolated blisters	Oral enanthema and oral ulcers, multiple erythematous papules, and itchy and painful blisters
**Location of Skin Lesions**	Scalp, face, chest, hands, and feet (Fig. 1)	Scalp, face, auricular pavilions, hands, thighs, and feet (Fig. 2)	Oral mucosa, back of the tongue, and hard palate. Armpits, elbows, hands, and feet (Fig. 3)
**Systemic Symptoms**	Fever, odynophagia, and general malaise	Fever, myalgia, and bilateral orchitis	Odynophagia
**Presumptive Diagnosis**	Syphilis and HFMD	Syphilis, HFMD, and blistering diseases	Syphilis, HFMD, milker's nodules, blistering diseases, and Kaposi's chickenpox-like rash
**Laboratory**	No changes	Leukocytosis (12000/mm^3^)	Leukocytosis (13100/mm^3^)
**Serologies**	VDRL and HIV non-reactive	HCV, HBV, VDRL, HIV, CMV, EBV, and *Parvovirus* non-reactive	HCV, HBV, VDRL, HIV, CMV, EBV, HSV, HZV, *Mycoplasma,* and *Parvovirus* non-reactive.
	IgM for *Coxsackie* virus B1-B6 reactive	IgM for *Coxsackie* virus B1-B6 reactive	IgG for *Coxsackie* virus with 4× titer increase 15 days later
**Skin Biopsy**	–	Compatible with viral infection, probable HFMD (Fig. 4)	Compatible with viral infection, probable HFMD
**Diagnosis**	Serological	Serological and histological	Serological and histological
**Treatment**	NSAIDS	NSAIDS and wet treatments of denuded blisters	NSAIDS and gargles with lidocaine
**Clinical Progress**	Resolution in 10 days	Resolution in 15 days	Resolution in 20 days
**Complications**	–	Onycholysis on hands and feet (2 months later)	–

HFMD, Hand, Foot, and Mouth Disease; HCV, Hepatitis C virus; HBV, Hepatitis B virus; VDRL, Venereal Disease Research Laboratory; HIV, Human Immunodeficiency Virus; CMV, Cytomegalovirus; EBV, Epstein-Barr Virus; HSV, Herpes Simplex Virus; HZV, Herpes Zoster Virus.

## Discussion

HFMD is an uncommon viral disease in adults as a result of cross-immunity with other enteroviruses and immunological memory. However, an increase in cases with atypical clinical presentation and a troublesome diagnosis has been observed in this age group in recent years.^5^

Unlike international publications, the cases reported in the present publication were caused by *Coxsackievirus B1-B6*.

The clinical manifestations of HFMD in adults are different from the typical manifestations seen during childhood since they may appear as blisters and purpuric skin rashes being more frequently serious and extensive.^2^, ^3^, ^4^, ^5^, ^6^

In the oral cavity, HFMD may appear as lesions ranging from enanthema with blisters to ulcers that may affect any area of the oral mucosa. Therefore, it must be differentiated from other diseases that affect the oral cavity, such as acute herpetic gingivostomatitis (AHGS), recurrent aphthous stomatitis (RAS), herpes simplex, and herpangina. In AHGS and RAS, the lesions tend to be bleeding ulcers that affect the gums, tongue, hard palate, and, in some cases, the pharynx. Herpangina appears as a painful papulo-vesicular enanthema, which can progress to small grayish-yellow ulcers with erythematosus borders. In most cases, it compromises the soft palate, tonsils, and posterior pharynx and is usually preceded by a high fever. However, none of these diseases concurrently affect the hands and feet as HFMD does.^1^, ^2^, ^3^, ^4^, ^5^, ^7^

The usual papulo-vesicular rash in HFMD involves the hands (backs of fingers, interdigital area, palms) and feet (tops of toes, lateral edges of feet, soles, and heels). The rash is usually asymptomatic, and it appears after the oral lesions. These manifestations must pose the differential diagnosis with shingles, chickenpox, Gianotti-Crosti syndrome, Orf nodules, and syphilis. As seen in the present study’s patients, atypical skin manifestations spread beyond the classic locations of HFMD, affecting the backs of the hands and tops of the feet, limbs, trunk, buttocks, peribucal region, and scalp. Shingles blisters are distinguished by their metameric distribution in clusters and for being painful. In chickenpox, skin involvement is characterized by its cephalocaudal distribution, with itchy lesions in different stages of evolution. In the Gianotti-Crosti syndrome, the rash starts suddenly with monomorphic edematous papules, skin-colored or reddish-pink papulo-vesicles symmetrically distributed on the face, buttocks, and limb extensor surfaces, and it usually does not appear on the trunk. Orf nodules are mainly located on the hands presenting as erythematosus maculopapular lesions, which progress to target blisters and, eventually, to bluish nodules that may ulcerate. In secondary syphilis lesions, though there is palmoplantar involvement, the trunk may also be affected, and it usually does not present blisters.^1^, ^2^, ^3^, ^4^, ^5^, ^6^, ^7^, ^8^

The diagnosis of HFMD is typically based on clinical grounds. However, in adults and in cases of atypical manifestations, complementary studies are generally required to allow for viral confirmation and to rule out other differential diagnoses.^2^, ^3^, ^4^, ^5^, ^6^

IgM and IgG serologies must be requested on days 0 and 15 to assess seroconversion. Viral cultures have low sensitivity.^5^

A skin biopsy is not necessary for an accurate diagnosis, but it is very useful to rule out differential diagnoses. In HFMD histology, intraepidermal blisters with neutrophil content, mononuclear cells, and eosinophilic proteinaceous material are observed. Spongiosis, reticular degeneration of the granular layers and the upper part of the stratum spinosum, keratinocyte mass necrosis, neutrophil exocytosis, and basal-layer hydropic degeneration are also observed. These findings help perform a diagnosis by correlating them with the clinical presentation and the remaining laboratory tests. DNA amplification through RT-PCR is the preferred method for an accurate diagnosis of an atypical disease, but this is only performed at high-tech laboratories and is not available at the present study’s institution.^3^, ^5^

Treatment is symptomatic. A patient diagnosed with HFMD is potentially contagious as long as they present skin lesions. Therefore, they must be excluded from the group and school activities until fever and skin, and mucosal lesions have disappeared. Furthermore, as a preventive measure, it is also recommended not to share objects or utensils and to meticulously wash hands to stop the spread of the disease.^3^

This entity must be recognized both in children and adults to avoid unnecessary studies and treatments.^3^, ^4^, ^5^

## Financial support

None declared.

## Authors' contributions

Anama Di Prinzio: Critical literature review; data collection, analysis, and interpretation; effective participation in research orientation; intellectual participation in propaedeutic and/or therapeutic management of studied cases; intellectual participation in propaedeutic and/or therapeutic management of studied cases; study conception and planning.

Dolores Pilar Bastard: Critical literature review; study conception and planning.

Ana Clara Torre: Effective participation in research orientation; intellectual participation in propaedeutic and/or therapeutic management of studied cases; intellectual participation in propaedeutic and/or therapeutic management of studied cases; manuscript critical review; study conception and planning.

Luis Daniel Mazzuoccolo: Approval of the final version of the manuscript; manuscript critical review.

## Conflicts of interest

None declared.

## References

[1] Mirand A., Peigue-Lafeuille H. (2017). Clinical characteristics and course of hand, foot, and mouth disease. Arch Pediatr..

[2] Stewart C.L., Chu E.Y., Introcaso C.E., Schaffer A., James W.D. (2013). Coxsackievirus A6–induced hand foot-mouth disease. JAMA Dermatol..

[3] Omaña-Cepeda C., Martínez-Valverde A., Sabater-Recolons M.M., Jané-Salas E., Marí-Roig A., López-López J. (2016). A literature review and case report of hand, foot, and mouth disease in an immunocompetent adult. BMC Res Notes..

[4] Shin J.U., Oh S.H., Lee J.H. (2010). A case of hand-foot-mouth disease in an immunocompetent adult. Ann Dermatol..

[5] Bennesch M.A., Pardal P.F., Salvaneschi B. (2017). Enfermedad mano-pie-boca del adulto, emergencia del Coxsackie A6. Dermatol Argent..

[6] Kaminska K., Martinetti G., Lucchini R., Kaya G., Mainetti C. (2013). Coxsackievirus A6 and hand, foot and mouth disease: three case reports of familial child-to-immunocompetent adult transmission and a literature review. Case Rep Dermatol..

[7] Yao X., Bian L.L., Lu W.W., Li J.X., Mao Q.Y., Wang Y.P. (2017). Epidemiological and etiological characteristics of herpangina and hand foot mouth diseases in Jiangsu, China, 2013-2014. Hum Vaccin Immunother..

[8] Panizzardi A.A., Luna P.C., Abad M.E., Vargas A., Plumet J., Casas J. (2018). Infección por parapoxvirus: orf y nódulo de los ordeñadores. Dermatol Arg..

